# Differences in growth in prepubertal children with definite growth hormone deficiency, short stature unresponsive to stimulation tests, and idiopathic short stature treated with recombinant human growth hormone: a retrospective study

**DOI:** 10.3389/fendo.2025.1628072

**Published:** 2025-10-15

**Authors:** Gianluca Tamaro, Maria Andrea Lanzetta, Martin Ove Carlsson, Daria La Torre, Gianluca Tornese

**Affiliations:** ^1^ Institute for Maternal and Child Health IRCCS “Burlo Garofolo”, Trieste, Italy; ^2^ University of Trieste, Trieste, Italy; ^3^ Pfizer Inc., New York, NY, United States; ^4^ Pfizer srl, Rome, Italy

**Keywords:** short stature, growth hormone, near adult height, prepubertal, idiopathic short stature, short stature unresponsive to stimulation tests, stimulation tests

## Abstract

**Introduction:**

Growth hormone stimulation tests are crucial in diagnosing growth hormone deficiency (GHD) in children; however, their limited reliability and inconsistent thresholds pose diagnostic challenges. A proposed subclassification distinguishes definite GHD (dGHD), short stature unresponsive to stimulation (SUS), and idiopathic short stature (ISS). This study aims to assess whether these categories are distinguishable at baseline and differ in response to recombinant human growth hormone (rhGH) therapy, particularly in terms of near adult height (NAH) outcomes.

**Methods:**

This retrospective cohort study analyzed data from 3,939 prepubertal children in the KIGS (Pfizer International Growth Database) who received rhGH therapy and reached NAH. Patients were classified into three groups: dGHD (GH peak <8 ng/mL with identifiable genetic, functional, or anatomical causes), SUS (GH peak <8 ng/mL without an identifiable cause), and ISS (GH peak ≥8 ng/mL). Multivariable regression analyses assessed the association of various factors with NAH outcomes.

**Results:**

Children with SUS showed baseline differences from those with dGHD but responded similarly to rhGH, with a height SDS increase of 0.13 for SUS and 0.12 for dGHD. In contrast, ISS children exhibited a smaller response (0 SDS increase). At the end of rhGH treatment, 74% of dGHD and SUS patients achieved a normal height (≥-2 SDS), compared to 65% of ISS patients. The most significant predictors of NAH included height at treatment initiation and mid-parental height, particularly in ISS patients.

**Conclusion:**

Despite initial differences, children with SUS responded similarly to rhGH as dGHD patients, while ISS patients had a less favorable response. These findings support the importance of subclassifying short stature conditions to refine diagnostic processes, enhance treatment approaches, and improve growth outcome predictions.

## Introduction

The diagnosis of growth hormone deficiency (GHD) relies on a combination of clinical, auxological, and laboratory criteria. Among these, an insufficient response to stimulation tests for endogenous growth hormone (GH) secretion represents the final step in confirming the diagnosis (1). However, it has long been recognized that stimulation tests face challenges in reproducibility and reliability, including false negatives even in children with genetically confirmed GHD (2, 3). Furthermore, there is considerable inconsistency in threshold values for diagnosing GHD across different countries, ranging from 3.3 ng/mL in Australia/New Zealand (4) to 10 ng/mL in Poland and the USA (5, 6). While in some countries (e.g., USA) the use of recombinant human growth hormone (rhGH) is permitted for idiopathic short stature (ISS), in most countries a diagnosis of GHD is required to access treatment. Given the clinical, economic, and ethical implications of GH therapy (7, 8), refining our understanding of the population currently classified as GHD is essential.

Based on these observations, it has been proposed that among children with unsatisfactory responses to GH stimulation tests, only those with an identifiable anatomical, functional, or genetic cause should be classified as definite GHD (dGHD). In contrast, others should be categorized as “short stature unresponsive to stimulation tests” (SUS) (9) rather than idiopathic GHD. One of the most important aspects of this classification is its role in guiding the future follow-up of these children. For instance, a child diagnosed with GHD has a greater than 5% risk of progressing to combined pituitary hormone deficiency (5) and a significantly increased likelihood of persistent GHD in adulthood (10).

In a previous study, we reported that dGHD and SUS patients exhibit distinct characteristics, with only dGHD patients showing features consistent with the etiopathogenesis of GHD, such as lower pre-treatment IGF-1 levels followed by a greater increase during therapy, higher BMI, and a higher prevalence of positive retesting at the end of treatment (11). As a matter of fact, lower baseline IGF-1 levels, followed by normalization after treatment, are indicative of GHD, reflecting the liver’s GH-stimulated secretion of IGF-1 (12). Moreover, GHD in children is often associated with mild to moderate overweight, which is considered a hallmark feature of the condition (13). Furthermore, organic GHD carries up to a 100% risk of persisting at retesting, compared to approximately one-third in cases without an organic cause (10).

Nevertheless, we found that rhGH supplementation was effective both in dGHD and in SUS in terms of improving near adult height (NAH) (11). Therefore, the SUS category should be distinguished from GHD to avoid labelling children with a condition that may not be definitively confirmed. Based on its poor response to stimulation tests and its positive response to GH treatment, SUS should also be distinguished from ISS and constitutional delay of growth and puberty (CDGP).

Building upon these findings, the present study aimed to further define the clinical and laboratory characteristics of SUS and dGHD patients using a large, multicenter international database of patients treated with rhGH. Specifically, the objective was to determine whether classification into dGHD, SUS, or ISS can predict the NAH of prepubertal children treated with rhGH.

## Materials and methods

This analysis was performed using the KIGS (Pfizer International Growth Database) observational dataset, which spans from its inception in 1987 until 2012. At the time of analysis, the KIGS dataset contained data on 83,803 children treated with rhGH.

In this study, we included all patients who had undergone a GH stimulation test, initiated rhGH treatment before puberty (Tanner stage B or G <2), and had reached near adult height (NAH). NAH was defined by a height velocity of less than 2 cm/year, an individual growth curve showing asymptotic growth toward adult height, and a bone age of at least 15 years, and treatment with GH for at least 5 years. Children with congenital GHD, neurosecretory dysfunction, Turner syndrome, Prader-Willi syndrome, other syndromes, and chronic renal insufficiency were excluded from the analysis. When neonatal data were available, patients born small for gestational age were also excluded.

At baseline, data on maximum GH peak during the stimulation test, sex, gestational age, weight and length at birth, and midparental height (MPH) were collected. Given that KIGS was an international registry, it seemed unlikely that homogeneous criteria for defining GHD were applied. Therefore, we established the Italian cut-off specifically for this study to ensure consistency and facilitate comparison with the previous report (11). Definite growth hormone deficiency (dGHD) was defined as a peak GH level of <8 ng/dL (14) and the presence of an identifiable genetic, functional, or anatomical cause. This included cases with a genetic diagnosis of isolated GHD (e.g., GH1, GHRHR, RNPC3), multiple pituitary hormone deficiency, or acquired damage (e.g., brain trauma, central nervous system infections, tumors of the hypothalamus or pituitary, cranial or total body irradiation, infiltrative diseases), or the presence of hypothalamic or pituitary abnormalities on MRI. Short stature unresponsive to stimulation tests (SUS) was defined as a peak GH level of <8 ng/dL without any identifiable genetic, functional, or anatomical cause (9). Idiopathic short stature (ISS) was defined as a peak GH level of ≥8 ng/dL. MPH SDS was calculated as follows: (father’s height SDS + mother’s height SDS) ÷ 1.61. This formula corrects for the correlation between parental heights due to assortative mating (15).

At the start of rhGH treatment, we collected data on chronological age, bone age, height SDS, IGF-1 SDS, height velocity (HV) in SDS, weight SDS, BMI SDS, and rhGH dose in mcg/kg/day. We also calculated bone age delay (chronological age − bone age), the prevalence of short stature (height <-2 SDS), and the difference between height and MPH in SDS. Bone age was determined by the treating clinician and/or radiologist through the Greulich-and-Pyle method (16).

In the KIGS registry, height and HV were converted to standard deviation scores (SDS) using height references for healthy children from Prader (17). Weight SDS was calculated using the normal population reference from Freeman (18), and BMI SDS was calculated using the normal population reference from Cole (19).

After 1 year of treatment, at the start of puberty, and at the last visit, we also calculated the changes in height and IGF-1 compared to the start of treatment (delta height and delta IGF-1, both in SDS), as well as the years of treatment duration.

Ethical Committee approval was not required for this study. The General Authorization to Process Personal Data for Scientific Research Purposes (Authorization no. 9/2014) by The Italian Data Protection Authority declared that retrospective archive studies using ID codes, which prevent data from being traced directly back to the data subject, do not need ethics approval (20).

Descriptive statistics were used to describe the data. Continuous variables were presented as medians (10th-90th percentiles) unless noted. Categorical data were expressed as percentages (%). For the pairwise comparisons of continuous variables between groups, the Mann-Whitney U test was used. The chi-square test was used to compare categorical variables between the groups.

Statistical significance was considered for p-values <0.05. To maintain an overall significance level of 0.05, we used the Bonferroni correction method in **Tables 1**–**3**, and the bivariate correlation in **Table 4** was calculated by Spearman correlation.

**Table 1:** Baseline characteristics.

Background/Start of GH	SUS				dGHD				ISS				P-value (dGHD vs. SUS)	P-value (dGHD vs. ISS)	P-value (SUS vs. ISS)
	N	Median	10^th^ pctl	90^th^ pctl	N	Median	10^th^ pctl	90^th^ pctl	N	Median	10^th^ pctl	90^th^ pctl			
Birth weight (SDS)	1173	-0.67	-2.24	0.83	1255	-0.40	-1.97	1.03	1131	-0.77	-2.19	0.66	<0.0001	<0.0001	0.0299
Birth length (SDS)	900	-0.51	-2.20	1.18	901	-0.19	-2.01	1.63	925	-0.72	-2.36	0.78	<0.0001	<0.0001	0.0026
Gestational age (weeks)	1196	40.00	36.00	41.00	1291	40.00	36.00	41.00	1146	40.00	36.00	41.00	0.0043	<0.0001	0.7010
MPH (SDS)	1240	-1.20	-2.78	0.34	1298	-0.65	-2.27	0.82	1166	-1.33	-2.92	0.05	<0.0001	<0.0001	0.0007
Max GH peak (ng/dL)	1300	5.15	1.73	7.60	1401	2.70	0.60	6.80	1238	10.60	8.50	23.10	<0.0001	<0.0001	<0.0001
Chronological age (years)	1300	8.50	4.63	11.69	1401	8.13	3.72	11.72	1238	8.84	5.29	11.75	0.0024	<0.0001	0.0009
Bone age (years)	445	6.08	2.50	10.00	515	5.70	2.00	10.00	468	6.83	3.00	10.00	0.0382	<0.0001	0.0734
Bone age delay (years)	445	2.11	0.54	3.57	515	2.02	0.33	4.20	468	2.04	0.49	3.67	1.0	1.0	1.0
Height (cm)	1300	113.70	90.50	130.80	1401	112.30	85.00	132.60	1238	116.25	96.50	130.80	0.0944	<0.0001	0.0002
Height (SDS)	1300	-3.12	-4.74	-2.12	1401	-3.26	-5.14	-1.34	1238	-3.08	-4.29	-2.11	1.0	0.0467	0.0561
Height - MPH (SDS)	1240	-1.95	-3.87	-0.51	1298	-2.48	-4.65	-0.67	1166	-1.71	-3.31	-0.22	<0.0001	<0.0001	<0.0001
IGF-I (ng/dL)	265	87.00	29.00	182	202	58.50	12.00	160.00	302	92.00	27.80	187.00	<0.0001	<0.0001	1.0
IGF-I (SDS)	265	-1.91	-3.28	-0.63	201	-2.50	-4.29	-0.72	302	-1.81	-3.15	-0.47	<0.0001	<0.0001	0.4279
Height velocity (cm/year)	616	4.43	2.77	6.26	674	3.92	1.78	6.62	636	4.56	3.06	6.27	<0.0001	<0.0001	0.2053
Height velocity (SDS)	614	-1.58	-3.81	0.38	667	-2.38	-5.29	0.03	635	-1.37	-3.44	0.38	<0.0001	<0.0001	0.0190
Weight (kg)	1300	19.73	12.40	30.00	1401	20.50	11.10	34.00	1238	21.00	13.50	28.60	0.3309	1.0	0.0060
Weight (SDS)	1300	-2.20	-4.06	-0.79	1401	-1.82	-4.30	0.44	1238	-2.21	-3.60	-0.86	-<0.0001	-<0.0001	1.0
BMI (kg/m^2^)	1300	15.56	13.78	18.63	1401	16.43	14.07	20.83	1238	15.55	13.79	18.02	-<0.0001	-<0.0001	0.9817
BMI (SDS)	1300	-0.35	-1.79	1.13	1401	0.20	-1.57	1.87	1238	-0.45	-1.81	0.84	-<0.0001	-<0.0001	0.0654
rhGH dose (microg/kg/day)	1300	30.23	21.06	42.92	1401	27.66	18.74	40.80	1238	30.30	21.81	42.02	-<0.0001	-<0.0001	1.0

The Mann-Whitney U test was used for pairwise comparisons, and the resulting p-values were adjusted using the Bonferroni correction method to control for multiple comparisons.

BMI, Body Mass Index; dGHD, definite Growth Hormone Deficiency; GH, Growth Hormone; IGF-1, Insulin-like Growth Factor 1; ISS, Idiopathic Short Stature; MPH, Mid-Parental Height; pctl, percentile; rhGH, recombinant human Growth Hormone; SDS, Standard Deviation Score; SUS, Short stature Unresponsive to Stimulation tests.

**Table 2:** Follow-up data.

	SUS				dGHD				ISS				P-value (dGHD vs. SUS)	P-value (dGHD vs. ISS)	P-value (SUS vs. ISS)
	N	Median	10^th^ pctl	90^th^ pctl	N	Median	10^th^ pctl	90^th^ pctl	N	Median	10^th^ pctl	90^th^ pctl			
First year on rhGH															
Chronological age (years)	1300	9.50	5.63	12.70	1401	9.12	4.75	12.71	1238	9.82	6.21	12.74	0.0021	<0.0001	0.0010
Bone age (years)	636	7.30	3.50	11.00	590	7.00	2.87	11.00	634	7.83	4.00	11.00	0.0759	<0.0001	0.0492
Bone age delay (years)	636	1.94	0.34	3.60	590	1.95	0.08	3.87	634	1.75	0.19	3.41	1.0	0.0269	0.0492
IGF-I (ng/dL)	108	209.00	85.00	410.00	88	202.00	40.00	487.80	127	198.00	77.00	369.00	1.0	1.0	1.0
IGF-I (SDS)	265	-1.91	-3.28	-0.63	201	-2.50	-4.29	-0.72	302	-1.81	-3.15	-0.47	1.0	1.0	1.0
Delta IGF SDS (vs. start)	78	1.51	-0.27	2.90	59	1.80	-0.52	3.2	83	1.21	0.10	2.42	0.8548	0.0285	0.2174
Height (cm)	1300	121.90	100.75	139.00	1401	121.30	95.50	141	1238	123.80	104.50	138.20	0.3237	<0.0001	0.0093
Height (SDS)	1300	-2.4	-3.86	-1.45	1401	-2.38	-4.27	-0.72	1238	-2.48	-3.64	-1.48	0.2310	0.0759	1.0
Height - MPH (SDS)	1240	-1.26	-2.97	0.12	1298	-1.67	-3.63	-0.01	1166	-1.07	-2.72	0.27	<0.0001	<0.0001	0.0004
Height velocity (cm/year)	1300	8.39	6.14	11.52	1401	8.85	5.79	12.99	1238	7.79	5.88	10.15	<0.0001	<0.0001	<0.0001
Height velocity (SDS)	1300	3.90	0.50	7.69	1401	3.92	-0.27	8.94	1238	3.08	0.11	6.29	1.0	<0.0001	<0.0001
Delta height SDS (vs. start)	1300	0.68	0.27	1.34	1401	0.74	0.12	1.67	1238	0.59	0.23	1.01	0.0344	<0.0001	<0.0001
Weight (kg)	1287	23.10	14.60	34.90	1388	23.85	13.80	38.80	1226	24.00	16.00	33.20	0.4620	1.0	0.0500
Weight (SDS)	1287	-1.66	-3.37	-0.35	1388	-1.34	-3.47	0.64	1226	-1.77	-3.13	-0.47	<0.0001	<0.0001	0.2934
BMI (kg/m^2^)	1287	15.72	13.85	18.73	1388	16.27	13.96	20.91	1226	15.75	13.85	18.40	<0.0001	<0.0001	1.0
BMI (SDS)	1287	-0.45	-1.78	0.89	1388	-0.08	-1.75	1.75	1226	-0.47	-1.79	0.76	<0.0001	<0.0001	0.1374
rhGH dose (microg/kg/day)	1300	30.02	21.26	42.35	1401	27.47	19.08	40.48	1238	30.29	21.60	42.18	<0.0001	<0.0001	<0.0001
At puberty															
Chronological age (years)	434	12.52	10.79	14.26	577	13.20	10.92	15.82	416	12.36	10.81	14.37	<0.0001	0.0001	1.0
Bone age (years)	140	11.15	9.50	13.23	184	11.54	9.50	13.50	151	11.00	9.00	13.25	1.0	0.0558	0.3800
Bone age delay (years)	140	1.03	-0.19	2.35	184	1.68	-0.13	4.23	151	1.40	-0.26	2.92	<0.0001	0.0284	0.0866
IGF-I (ng/dL)	51	340.70	140.00	509.00	63	288.41	130.00	491.00	62	351.00	166.00	466.80	1.0	0.2204	1.0
IGF-I (SDS)	51	-0.25	-2.82	1.00	63	-0.97	-2.99	0.98	62	-0.30	-2.19	1.04	0.2636	0.0266	1.0
Delta IGF SDS (vs. start)	30	1.70	0.32	3.49	34	1.50	-0.31	3.26	27	2.11	-0.02	3.96	1.0	0.7014	1.0
Height (cm)	434	141.8	132.50	152.50	577	147.00	133.40	161.20	416	140.45	130.10	151.00	<0.0001	<0.0001	0.0102
Height (SDS)	434	-1.14	-2.34	0.04	577	-0.87	-2.39	0.64	416	-1.36	-2.41	-0.36	<0.0001	<0.0001	0.0002
Height - MPH (SDS)	419	-0.02	-1.47	1.14	539	-0.35	-1.83	1.18	383	-0.13	-1.85	1.22	0.0168	0.2603	0.9939
Height velocity (cm/year)	412	6.21	4.72	8.16	526	5.89	3.71	8.04	385	6.15	4.44	7.73	0.0006	0.0333	0.7019
Height velocity (SDS)	412	0.99	-2.65	3.96	522	0.68	-3.39	5.12	385	0.98	-2.81	4.31	1.0	0.6354	1.0
Delta height SDS (vs. start)	434	1.89	1.11	3.58	577	2.28	0.98	4.46	416	1.63	0.92	2.64	<0.0001	<0.0001	<0.0001
Weight (kg)	433	34.60	28.00	47.10	575	40.20	30.50	59.60	416	33.00	27.00	42.90	<0.0001	<0.0001	0.0008
Weight (SDS)	433	-0.91	-2.32	0.58	575	-0.49	-2.49	1.48	416	-1.25	-2.57	0.14	<0.0001	<0.0001	<0.0001
BMI (kg/m^2^)	433	17.11	15.06	22.25	575	18.60	15.75	25.10	416	16.89	14.78	20.42	<0.0001	<0.0001	0.0843
BMI (SDS)	433	-0.35	-1.78	1.39	575	0.19	-1.52	2.03	416	-0.51	-1.96	0.77	<0.0001	<0.0001	0.0382
rhGH dose (microg/kg/day)	435	30.75	21.18	43.67	579	26.66	16.44	38.12	417	32.40	22.39	48.05	<0.0001	<0.0001	0.0165
Treatment duration (years)	434	4.27	2.31	8.15	577	5.27	2.59	10.00	416	3.72	2.30	6.83	<0.0001	<0.0001	0.0036

The Mann-Whitney U test was used for pairwise comparisons, and the resulting p-values were adjusted using the Bonferroni correction method to control for multiple comparisons.

BMI, Body Mass Index; dGHD, definite Growth Hormone Deficiency; GH, Growth Hormone; IGF-1, Insulin-like Growth Factor 1; ISS, Idiopathic Short Stature; MPH, Mid-Parental Height; rhGH, recombinant human Growth Hormone; SDS, Standard Deviation Score; SUS, Short stature Unresponsive to Stimulation tests.

**Table 3:** Characteristics at last visit.

At NAH	SUS				dGHD				ISS				P-value (dGHD vs. SUS)	P-value (dGHD vs. ISS)	P-value (SUS vs. ISS)
	N	Median	10^th^ pctl	90^th^ pctl	N	Median	10^th^ pctl	90^th^ pctl	N	Median	10^th^ pctl	90^th^ pctl			
Chronological age (years)	1300	17.12	15.14	19.05	1401	17.80	15.86	20.35	1238	17.16	15.02	19.13	<0.0001	<0.0001	1.0
Bone age (years)	222	16.00	14.00	17.50	156	15.50	14.00	18.00	207	16.00	14.00	17.00	0.0941	0.3851	1.0
Bone age delay (years)	222	0.77	-0.61	2.62	156	1.60	-0.32	4.19	207	0.89	-0.55	2.59	<0.0001	<0.0001	1.0
IGF-I (ng/dL)	237	306.00	157.00	586.00	264	226.50	56,00	458.00	269	329.00	186.30	537.00	<0.0001	<0.0001	0.6833
IGF-I (SDS)	237	-1.72	-3.75	0.83	264	-2.72	-5.73	-0.03	269	-1.35	-3.22	0.67	<0.0001	<0.0001	0.5793
Delta IGF SDS (vs. start)	98	0.44	-1.48	3.23	91	0.06	-2.22	2.12	104	0.37	-1.56	2.35	0.0909	0.4697	1.0
Height (cm)	1300	164.30	150.00	175.50	1401	164.80	149.70	178.20	1238	162.05	147.90	172.80	0.0325	<0.0001	<0.0001
Height (SDS)	1300	-1.28	-2.89	-0.01	1401	-1.06	-3.00	0.64	1238	-1.59	-3.21	-0.25	<0.0001	<0.0001	<0.0001
Height - MPH (SDS)	1240	-0.05	-1.58	1.18	1298	-0.34	-2.19	1.19	1166	-0.13	-1.85	1.15	<0.0001	0.0017	0.2283
Height velocity (cm/year)	1090	1.59	0.18	3.71	1161	1.10	0.00	3.39	1035	1.58	0.00	3.55	<0.0001	<0.0001	1.0
Height velocity (SDS)	1036	0.35	-1.94	2.94	991	0.44	-1.83	3.68	966	0.42	-2.11	3.00	0.1446	0.3349	1.0
Delta height SDS (vs. start)	1300	1.80	0.72	3.30	1401	2.08	0.17	4.16	1238	1.51	0.38	2.52	0.0012	<0.0001	<0.0001
Weight (kg)	1270	56.00	43.00	72.25	1373	59.50	43.90	84.70	1215	53.00	41.6	68.40	<0.0001	<0.0001	<0.0001
Weight (SDS)	1270	-0.69	-2.31	0.9	1373	-0.29	-2.47	2.06	1215	-1.03	-2.59	0.51	<0.0001	<0.0001	<0.0001
BMI (kg/m^2^)	1270	20.70	17.70	25.70	1373	21.94	17.97	29.54	1215	20.28	17.47	24.51	<0.0001	<0.0001	<0.0001
BMI (SDS)	1270	0.02	-1.45	1.55	1373	0.34	-1.55	2.30	1215	-0.15	-1.58	1.27	<0.0001	<0.0001	<0.0001
rhGH dose (microg/kg/day)	1300	29.81	21.41	41.31	1401	26.46	18.88	36.56	1238	31.28	22.30	45.65	<0.0001	<0.0001	<0.0001
Treatment duration (years)	1300	7.98	5.49	11.83	1401	8.99	5.87	13.82	1238	7.46	5.46	11.00	<0.0001	<0.0001	<0.0001

The Mann-Whitney U test was used for pairwise comparisons, and the resulting p-values were adjusted using the Bonferroni correction method to control for multiple comparisons.

BMI, Body Mass Index; dGHD, definite Growth Hormone Deficiency; GH, Growth Hormone; IGF-1, Insulin-like Growth Factor 1; ISS, Idiopathic Short Stature; MPH, Mid-Parental Height; NAH, near adult height; rhGH, recombinant human Growth Hormone; SDS, Standard Deviation Score; SUS, Short stature Unresponsive to Stimulation tests.

**Table 4:** Correlation coefficients between different variables and height in standard deviation score (SDS) at near adult height (NAH) for children with definite growth hormone deficiency (GHD), short stature unresponsive to stimulation tests (SUS) or idiopathic short stature (ISS).

Height SDS at NAH	dGHD			SUS			ISS		
	N	R	P value	N	R	P value	N	R	P value
Birth weight (SDS)	1255	0.220	<0.0001	1173	0.250	<0.0001	1131	0.262	<0.0001
Birth length (SDS)	901	0.245	<0.0001	900	0.241	<0.0001	925	0.225	<0.0001
Gestational age (weeks)	1291	0.095	0.0006	1196	0.071	0.0132	1146	0.051	0.0803
MPH (SDS)	1298	0.468	<0.0001	1240	0.544	<0.0001	1166	0.462	<0.0001
Height at start (SDS)	1401	0.396	<0.0001	1300	0.531	<0.0001	1238	0.625	<0.0001
Chronological age at start (SDS)	1401	-0.062	0.0193	1300	-0.061	0.0272	1238	0.021	0.4408
Bone age at start (SDS)	515	-0.101	0.0216	445	-0.014	0.7582	468	0.084	0.0680
Max GH peak (ng/dL)	1401	-0.191	<0.0001	1300	-0.027	0.3257	1238	-0.135	<0.0001
Height - MPH at start (SDS)	1298	0.002	0.9387	1240	-0.104	0.0002	1166	-0.035	0.2200
IGF-I at start (SDS)	201	-0.119	0.0914	265	-0.129	0.0348	302	-0.206	0.0003
Weight at start (SDS)	1401	0.320	<0.0001	1300	0.310	<0.0001	1238	0.322	<0.0001
BMI at start (SDS)	1401	0.164	<0.0001	1300	0.015	0.5730	1238	-0.035	0.2084
rhGH dose at start (microg/kg/day)	1401	0.024	0.3530	1300	0.170	<0.0001	1238	0.192	<0.0001
Treatment duration at NAH (years)	1401	0.136	<0.0001	1300	0.106	0.0001	1238	0.082	0.0035

GH, growth hormone; IGF-1, Insulin-like Growth Factor 1; MPH, midparental height; SDS, standard deviation score; rhGH, recombinant human growth hormone.

For the purposes of interpreting the magnitude of a correlation, r=0.10, r=0.30, and r=0.50 were considered small, medium, and large in magnitude, respectively (21). Furthermore, multivariable linear regression models for predicting height SDS at NAH in the SUS, dGHD, and ISS groups were developed and fitted by least squares and the REG procedure in SAS. A hierarchy of 10 predictive factors was derived using the all possible regression subsets approach, with Mallow’s C(p) criterion employed to order the predictive factors, as described by Weisberg and Cook (22, 23). This method involved evaluating all possible combinations of the candidate predictor variables to identify the subset of predictors that best balanced a model with low bias and a good balance between precision and parsimony. The 10 candidate predictors were: height SDS at rhGH start, birth weight SDS, birth length SDS, max GH peak, treatment duration, sex, rhGH dose, gestational age, age at start of GH, BMI SDS, modeled as individual effects.

An additional analysis was performed to explore interactions between the variables and diagnostic groups. No multiple comparison testing procedure was applied to select the regression coefficients. All statistical analyses were conducted with SAS^®^ version SAS, version 9.4 (SAS Institute).

## Results

The analysis included 3,939 children who had undergone GH stimulation tests, initiated rhGH treatment before puberty, and reached NAH. Of these, 1,401 were classified as having dGHD (36%), 1,300 as SUS (33%), and 1,238 as ISS (31%). All baseline characteristics, data after 1 year of treatment, at the start of puberty, and at NAH are reported in **Tables 1**-**3**; p-values are corrected for multiple comparisons.

### Baseline characteristics

As per definition, the median maximum GH peak was significantly higher in ISS (10.60 ng/dL [range 8.50;23.10]), but a significant difference was found also between dGHD (2.70 ng/dL [range 0.60;6.80]) and SUS (5.15 ng/dL [range 1.73;7.60]) (p<0.01 for all comparisons). The majority of patients in all groups were male, with a higher percentage in the SUS group (65.8%, p<0.01 vs. dGHD). Most patients were born at term, with a mean gestational age of 39 weeks, with no significant differences across groups. Birth weight and birth length SDS were significantly higher in the dGHD group compared to SUS and ISS, although neonatal anthropometric data were available for only a subset of patients. MPH and TH were also significantly higher in the dGHD group (p<0.01).

### At the start of rhGH treatment

**Figure 1:**
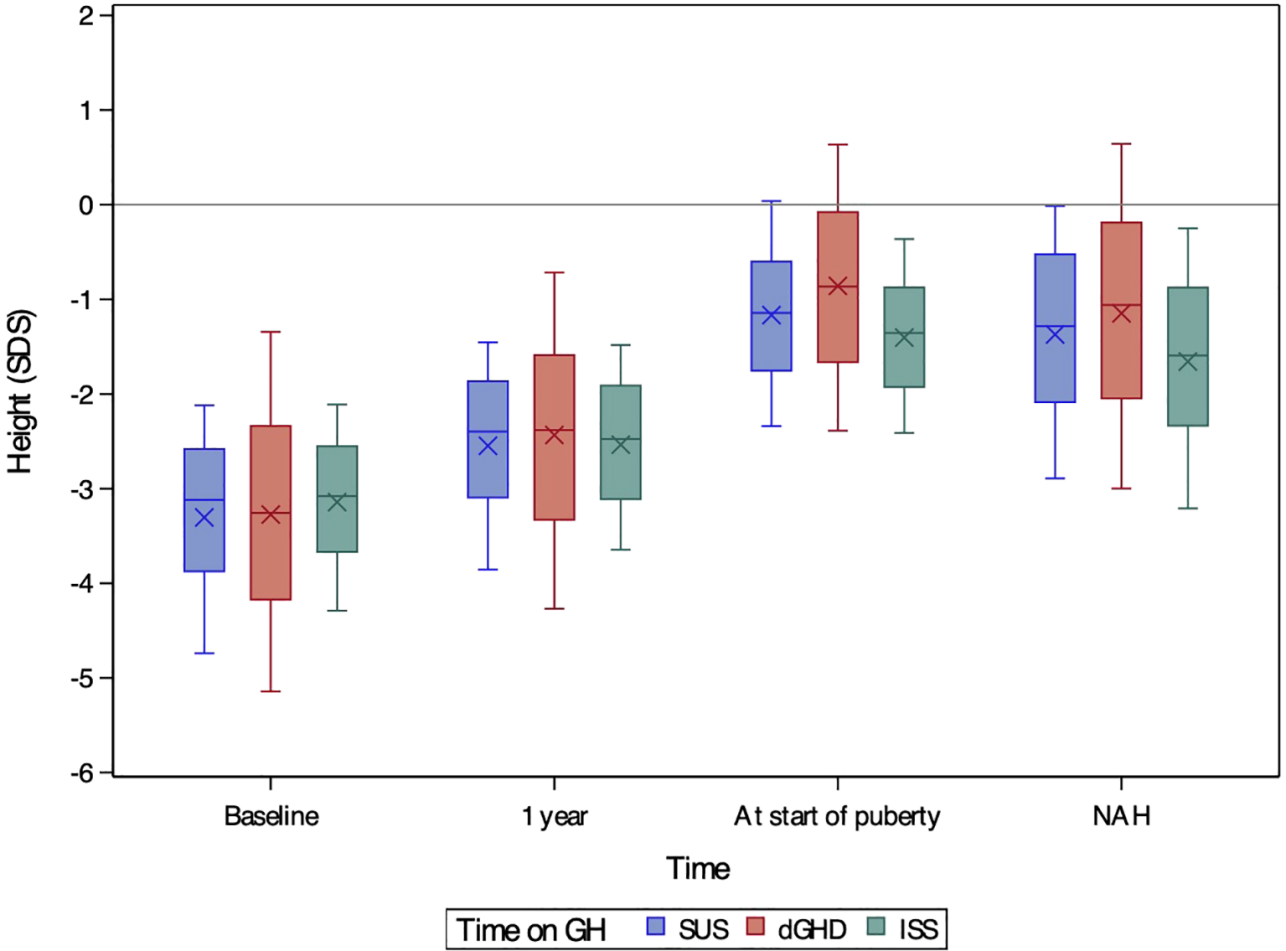
Boxplots of height SDS at baseline, after one year of treatment, at the onset of puberty, and at near-adult height (NAH) for children with short stature unresponsive to stimulation tests (SUS, blue), definite growth hormone deficiency (dGHD, red), and idiopathic short stature (ISS, green). The ‘X’ symbol represents the mean value, while the boxplots illustrate the median (central line), interquartile range (25th–75th percentile, box), and overall data distribution, excluding potential outliers.

At the start of rhGH treatment, patients in the dGHD group were significantly younger (8.13 years [range 3.72;11.72]) compared to SUS (8.50 years [range 4.63;11.69]) and ISS (8.84 years range 5.29;11.75]) (p<0.01 for all comparisons) (**Table 1**). Bone age delay was consistent across groups (approximately 2 years). Height SDS was similar between dGHD (-3.26 SDS [range -5.14;-1.34]) and SUS (-3.12 SDS [range -4.74;-2.12], p=0.09), with ISS showing slightly higher values than dGHD (-3.08 SDS [-4.29;-2.11], p<0.05) (**Figure 1**) and dGHD having a lower prevalence of short stature (82% in dGHD vs. 93% in both SUS and ISS, p<0.01). However, dGHD patients had a more pronounced difference between their height and MPH (-2.48 SDS [range -4.65;-0.67], p<0.01), a higher BMI SDS (0.20 SDS [range -1.57;1.87], p<0.01), and a lower starting rhGH dose (27.66 microg/kg/day [range 18.74;40.80], p<0.01) and a more compromised HV (-2.38 SDS [range -5.29;0.03], p<0.01) compared to both SUS and ISS (**Table 1**). Although IGF-1 data were available for only a subset of patients, levels were significantly lower in dGHD (-2.50 SDS [range -4.29; -0.72]) compared with SUS (-1.91 SDS [range -3.28; -0.63]) and ISS (-1.81 SDS [range -3.15; -0.47]) (p < 0.01 for both comparisons), while no significant difference was observed between SUS and ISS (p = 0.42).

### After 1 year of rhGH therapy

**Figure 2:**
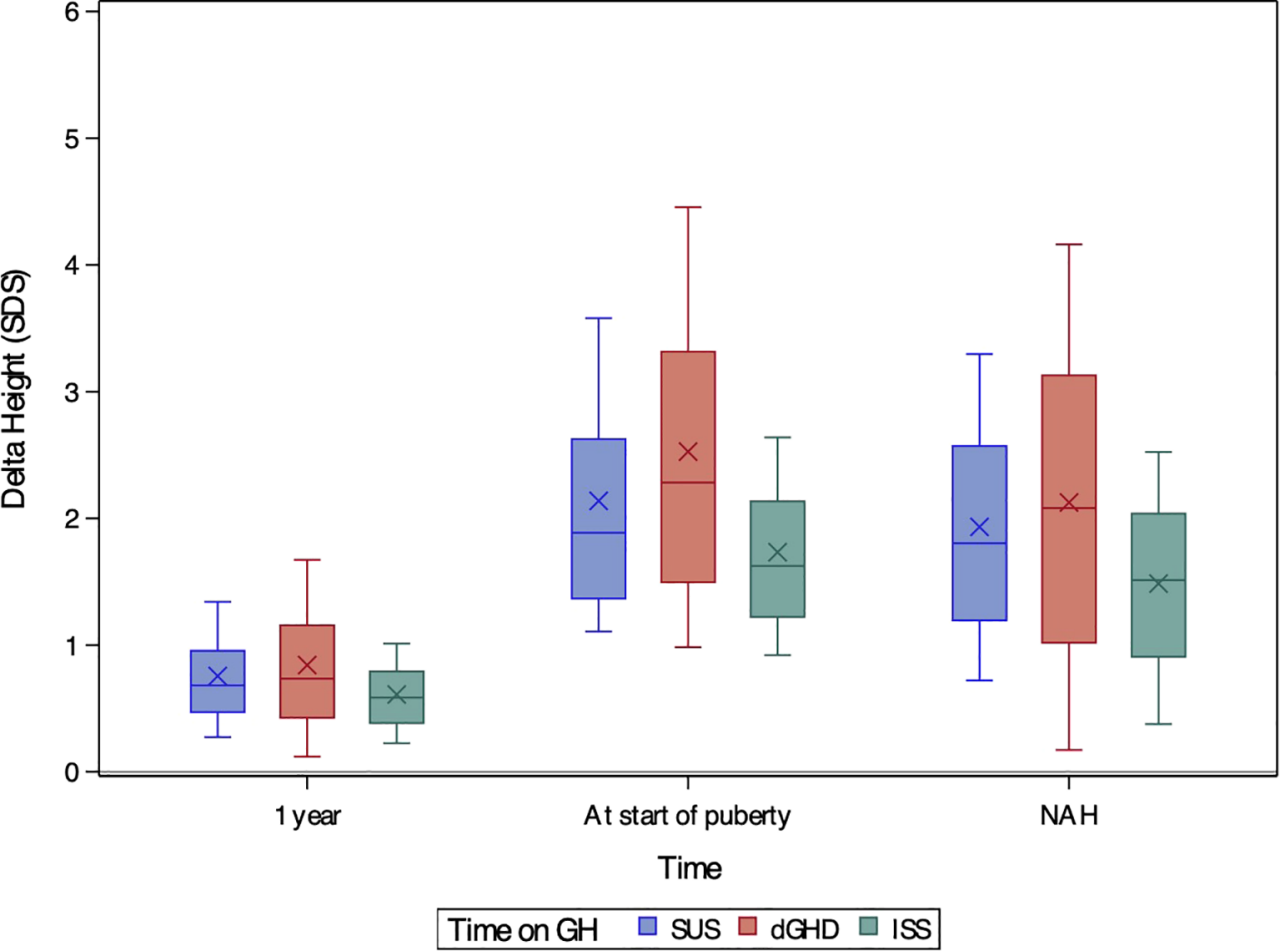
Boxplots of delta height SDS after one year of treatment, at the onset of puberty, and at near-adult height (NAH) for children with short stature unresponsive to stimulation tests (SUS, blue), definite growth hormone deficiency (dGHD, red), and idiopathic short stature (ISS, green). The ‘X’ symbol represents the mean value, while the boxplots illustrate the median (central line), interquartile range (25th–75th percentile, box), and overall data distribution, excluding potential outliers.

All groups showed significant improvements in height SDS after 1 year of treatment (**Figure 1B**), with height SDS gains being greatest in dGHD (+0.74 SDS [range 0.12;1.67]), followed by SUS (+0.68 SDS [range 0.27;1.34], p=0.03), and ISS (+0.59 SDS [range 0.23;1.01], p<0.01) (**Table 2**, **Figure 2**). IGF-1 levels also increased in all groups, with dGHD showing the largest improvement (+1.80 SDS [range -0.52;3.20]), although available data were limited. Height velocity (HV) increased significantly in all groups, with dGHD patients exhibiting the greatest improvement (3.92 SDS [range -0.27;8.94]).

### At puberty

Puberty onset was delayed in dGHD patients (13.20 years [range 10.92;15.82]), compared to SUS (12.52 years [range 10.79;14.26]) and ISS (12.36 years [range 10.81;14.37]) (p<0.01 for both comparisons) (**Table 2**). Bone age delay was more pronounced in dGHD (1.68 years [range -0.13;4.23), compared to SUS (1.03 years [range -0.19;2.35], p<0.01) and ISS (1.40 years [-0.26;2.92], p=0.03). Height SDS remained highest in the dGHD group (-0.87 SDS [range -2.39;0.64]) compared to SUS (-1.14 SDS [range -2,34;0.04]) and ISS (-1.36 SDS [range -2.41;-0.36]) (p<0.01 for all comparisons) (**Figure 1**).

### At last visit

At the last visit, the prevalence of short stature was similar in dGHD (26.2%) and SUS (26.6%), but higher in ISS (35.3%, p<0.01 vs. both groups) (**Table 3**). Final height SDS was highest in the dGHD group (-1.06 SDS [range -3.00;0.64]), followed by SUS (-1.28 SDS [range -2.89;-0.01]) and ISS (-1.59 SDS [range -3.21;-0.25]) (p<0.01 for all comparisons) (**Figure 1**). dGHD patients had the greatest overall improvement in height (+2.08 SDS [range 0.17;4.16]) compared to SUS (+1.80 SDS [range 0.72;3.30]) and ISS (+1.51 SDS [range 0.38;2.52]) (p<0.01 for all comparisons) (**Figure 2**). Despite these gains, the distance from MPH was still larger in dGHD (-0.34 SDS [range -2.19;1.19]) compared to SUS and ISS. BMI SDS remained higher in dGHD (0.34 SDS [range -1.55;2.30]) compared to SUS (0.02 SDS [range -1.45;1.55]) and ISS (-0.15 SDS [range -1.58;1.27]) (p<0.01 for all comparisons).

Age at NAH was higher in dGHD patients (17.80 years [range 15.86;20.35]) compared to SUS and ISS (p<0.01). Bone age delay persisted in the dGHD group (1.60 years [range -0.32;4.19]) but was less pronounced in SUS and ISS (p<0.01). dGHD patients also had a longer duration of rhGH treatment (8.99 years [range 5.87;13.82]), compared to SUS and ISS (p<0.01 for all comparisons), as well as a lower final rhGH dose (26.46 microg/kg/day [range 18.88;36.56]) compared to SUS (29.81 microg/kg/day [range 21.41;41.31]) and ISS (31.28 microg/kg/day [range 22.30;45.65]) (p<0.01 for all comparisons).

### Correlations

**Table 4** presents the correlations between various variables and height SDS at NAH across the dGHD, SUS, and ISS groups. Birth weight and birth length were positively correlated with height SDS at NAH across all groups, with r values ranging from 0.220 to 0.262 (p<0.01), indicating small to medium correlations. MPH showed a large correlation with height SDS in all groups, with r values ranging from 0.462 to 0.544 (p<0.01), suggesting that parental height plays a significant role in predicting NAH. Height at the start of treatment also exhibited a medium to large correlation with height SDS at NAH, with the strongest correlation found in the ISS group (r=0.625, p<0.01). Max GH peak was inversely correlated with height SDS at NAH, particularly in the dGHD (r=-0.191) and ISS (r=-0.135) groups (p<0.01), though these correlations were small in magnitude. Weight at the start of treatment was moderately correlated with NAH across all groups (ranging from r=0.310 to 0.322, p<0.01), reflecting a medium effect size. For BMI at the start, a small positive correlation was observed in the dGHD group (r=0.164, p<0.01), while no significant correlations were found in SUS and ISS. Treatment duration had a small correlation with NAH in all groups (ranging from r=0.082 to 0.136, p<0.05). Overall, the most influential predictors of height SDS at NAH were MPH and height at the start of treatment, both of which exhibited the strongest correlations, particularly in the ISS group.

### Multivariable regression analysis

Six variables (height SDS at rhGH start, birth weight SDS, max GH peak, treatment duration, sex, rhGH dose) were selected from Mallow’s C(p) criterion model selection approach described above and included in the selected regression model for predicting height SDS at NAH in the SUS, dGHD, and ISS groups. The equation for height SDS at NAH was as follows:

*Height SDS at NAH = -0.58 + [0.62 x Height SDS at rhGH start] + (0.14 x birth weight [SDS] + [-0.02 x Max GH peak at rhGH start] + (0.14 x treatment duration (yrs)) +[(-0.18 x [males=1; females=0])] + (0.01 x rhGH dose (mcg/kg/day)+ [(0.13 x [SUS=1; ISS=0])]+ [(0.12 x [dGHD=1; ISS=0])].*

This model had an R-square value equal to 0.38, indicating that 38% of the variance in Height SDS at could be explained by the six variables above. Height SDS at the start of rhGH treatment was the strongest predictor of NAH. Children in the ISS group had significantly lower NAH compared to both dGHD (-0.12, p=0.036) and SUS (-0.13, p=0.016), with no significant difference between dGHD and SUS (p>0.05). Additionally, boys had a significantly lower NAH than girls (-0.18, p<0.001). The model has 8 degrees of freedom for the predictors and 3,550 degrees of freedom for the error, based on a dataset that contained 380 missing values. The six selected variables based on the model selection approach were all significant at the 5% level of significance.

An additional analysis was performed to explore interactions between the variables and diagnostic groups. Significant two-way interactions were found for Height SDS at rhGH start and Max GH Peak across the SUS, dGHD, and ISS groups. For every one-unit increase in height SDS at rhGH start, the estimated height SDS at NAH increased by 0.97 for SUS, 0.70 for dGHD, and 1.06 for ISS. A constant effect was observed for Max GH Peak in the ISS group, while in the SUS and dGHD groups, each unit increase in max GH Peak was associated with an estimated decrease in height SDS at NAH of -0.06 and -0.09, respectively. Note that each regression coefficient is adjusted for all other variables in the model, implying that any differences reported here are adjusted differences that might deviate from those observed in the raw data.

**Figure 3:**
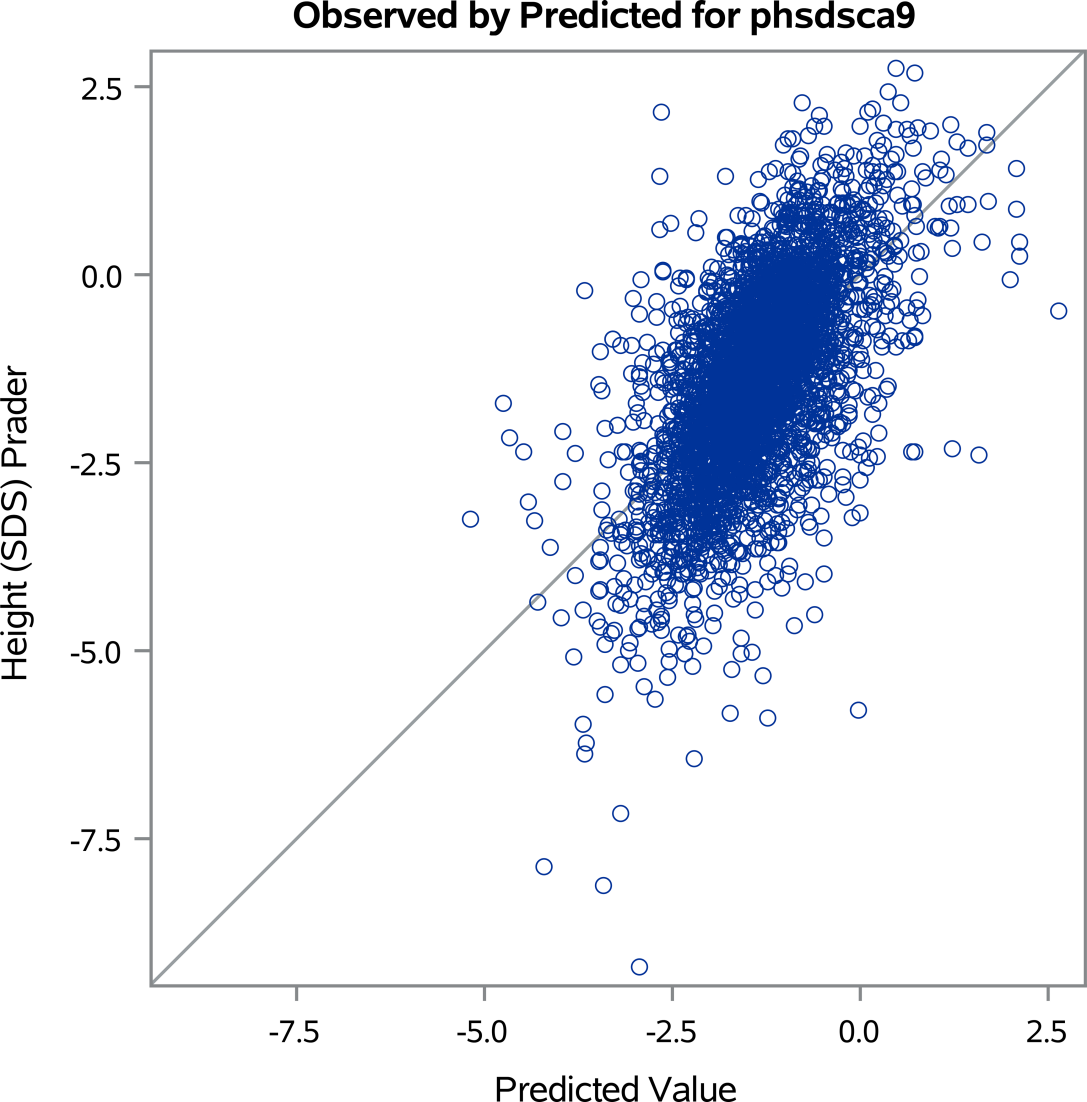
Predicted versus observed height standard deviation scores (SDS) at near adult height (NAH).

Visual diagnostics of the linear regression model were performed using plots generated by PROC REG in SAS. This analysis aimed to assess whether the model’s underlying assumptions of linearity, homoscedasticity, and normality of residuals were met; there were no systematic deviations detected. A plot (**Figure 3**) of predicted versus observed height SDS at NAH visually represents the agreement between a model’s predictions and the actual measurements. The R-squared value of 38% quantifies this relationship; the plot suggests that the predictions do not perfectly align with the observed values. Although 38% is not a strong R-squared value for a predictive model of Height SDS at NAH, it does suggest that the factors included in the model above have some ability to explain variations in Near Adult Height. However, it highlights the need to consider other contributing factors that are not collected in the KIGS registry for a more comprehensive understanding of a child’s potential adult height.

## Discussion

This study provides a comprehensive analysis of the differences among prepubertal children treated with rhGH and classified as dGHD, SUS, or ISS. By leveraging a large international cohort, we aimed to identify the most significant predictors of NAH and compare the response to prolonged rhGH therapy across these diagnostic groups.

The main finding of this study is that children with SUS have baseline differences from dGHD, but exhibited a similar response to rhGH treatment, in contrast to the distinct response seen in ISS patients. However, dGHD and SUS differ markedly in clinical, auxological, and biochemical profiles—such as GH peak levels, IGF-1 levels, BMI, and mid-parental height.

Regarding the long-term response to hormonal treatment, all three patient cohorts benefited from rhGH administration, resulting in an overall reduction in the prevalence of short stature. The prevalence of short stature at the start of treatment was similar between the SUS and ISS groups (93%), but lower in the dGHD group (82%). However, by the end of rhGH treatment, the prevalence of individuals achieving normal height was comparable between the dGHD and SUS groups, with 74% reaching a normal height, compared to only 65% of ISS patients who achieved this outcome. Patients with dGHD showed a higher NAH, achieving a median height of -1.06 SDS, compared to -1.28 SDS in SUS, with both groups significantly outperforming ISS patients, who reached -1.59 SDS. This difference does not appear to be related to rhGH dose, as children with dGHD experienced the greatest improvement in height SDS both during the first year of treatment (+0.74 SDS vs. +0.68 in SUS and +0.59 in ISS), while receiving significantly lower rhGH dose compared to SUS and ISS, and by the end of treatment (+2.08 SDS vs. +1.80 in SUS and +1.51 in ISS), when receiving higher rhGH doses. However, both SUS and ISS patients achieved a NAH closer to their genetic potential: the differences from MPH were -0.05 SDS in SUS and -0.13 SDS in ISS, compared to -0.34 SDS in dGHD. Ultimately, the multivariate analysis revealed that, when controlling for factors such as sex, birth weight, height at the start of treatment, GH peak at stimulation tests, treatment duration, and GH dose, the difference in NAH between dGHD and SUS was minimal, at only 0.01 SDS. On the contrary, ISS patients had a significantly lower NAH, with a difference of -0.13 SDS compared to dGHD and -0.12 SDS compared to SUS, further underscoring their lower overall growth potential in response to rhGH treatment, as already reported (24, 25). Nevertheless, in our cohort, the response of ISS children to rhGH treatment was found to be better than what has been reported in previous studies (26, 27). When considering bone age, it was found to be homogeneous throughout all categories at baseline; however, delay persisted during treatment and at NAH solely in the dGHD group. This is consistent with previous findings of bone age progression being more rapid in non-GHD patients treated with rhGH and that bone age progression in GHD patients reaches a plateau after an initial increase with replacement hormonal therapy (28, 29).

In our study, the most significant positive predictors of response to rhGH therapy, in terms of NAH, were height at the start of treatment and mid-parental height, particularly in the ISS group. It is well established that height is a polygenic trait, with normal adult height determined by the interaction of multiple factors. While nutritional, endocrinological, and social factors are important, up to 80-90% of adult height is genetically determined (30). Major gene mutations can lead to GHD or skeletal dysplasia, but polymorphisms, copy number variations, and milder mutations also play a critical role in regulating height (31). Therefore, it can be hypothesized that in children with ISS, who do not have a genetically determined GH deficiency, other inherited polygenic factors are likely key determinants of adult height, explaining the observed correlation with MPH.

It is noteworthy that SUS patients exhibited a similar response to rhGH treatment despite having distinct characteristics from children with dGHD, both at baseline and throughout treatment.

While both groups, by definition, had a GH peak <8 ng/mL, children with dGHD exhibited a significantly lower peak compared to those with SUS (2.70 vs. 5.15 ng/mL). It is well-known that GH stimulation tests suffer from issues with reproducibility, potentially yielding false positives in cases of syndromic short stature not related to GH deficiency (e.g., *SHOX* deficiency) (32), or false negatives in genetically confirmed GHD (3, 33). Nevertheless, Coutant et al. have reported cases of severe GHD due to identifiable genetic or anatomical abnormalities, or combined pituitary hormone deficiency (CPHD), where peak GH levels in stimulation tests were consistently <5 ng/mL, lower than in ‘idiopathic’ GHD cases (34). In addition to their intrinsic reproducibility issues, GH stimulation tests may also fail in children with otherwise normal growth, reflecting temporary or functional insufficiency of GH secretion rather than true GHD. Such conditions include CDGP, neurodevelopmental disorders such as ADHD (35, 36). These scenarios likely contribute to the high rate of failed stimulation tests reported in normally growing prepubertal children, with previous studies documenting failure rates ranging from about 23% to as high as 75% when conventional cut-offs are applied (37). This highlights the considerable risk of misclassification when relying solely on these tests.

In our study, we also observed a significant inverse correlation between GH peak and NAH, as previously documented (38). This suggests that individuals with lower GH peaks during stimulation tests may respond better to rhGH therapy. Notably, this correlation between GH peak and NAH was also evident in ISS patients, confirming prior concepts positioning ISS as a transitional state between GHD and GH insensitivity (39, 40). The homogeneity of this correlation throughout all categories of patients supports the hypothesis that SUS may represent an intermediate category between dGHD and ISS. This interpretation is further supported by the reduced rate of confirmation of pathological GH secretion upon retesting in SUS patients (11).

Compared to SUS patients, children with dGHD also had lower IGF-1 levels at diagnosis (-2.50 vs. -1.91 SDS). They also exhibited the best response to rhGH treatment after 1 year, with the highest increase in IGF-1 levels (+1.80 SDS vs. +1.51 in SUS and +1.21 in ISS, respectively). IGF-1 levels are known to be lower in organic GHD compared to idiopathic GHD (12), and our group previously demonstrated that dGHD patients have lower IGF-1 levels at diagnosis compared to SUS (11). Although IGF-1 levels can be influenced by various factors (such as malnutrition, pubertal stage, and age) (41), it is clear that they are highly dependent on GH secretion. It is important to note that confounding factors in our cohort were minimized, as all patients were prepubertal, and IGF-1 values were reported as SDS adjusted for sex and age. Moreover, ISS patients had higher IGF-1 levels, within the normal range, consistent with the different etiologies of short stature.

BMI in dGHD patients was higher compared to SUS patients, both at the start (0.20 vs. -0.35 SDS) and at the end of treatment (0.34 vs. 0.02 SDS), though still within the normal range. Previous studies have suggested that GHD in children is often associated with mild to moderate overweight (13). However, other research has reported that BMI tends to be within the average range, with no significant differences observed between organic and idiopathic GHD (42). From a metabolic perspective, GH acts as an anabolic hormone, promoting lipolysis, lipid uptake in skeletal muscle, and insulin synthesis (43). Rather than focusing solely on BMI, attention should be given to fat distribution patterns. However, our study did not assess body composition, so it is unclear whether the relative increase in BMI observed in dGHD patients was due to an increase in lean mass or fat mass (44).

With regard to birth weight and length, dGHD patients had a history of higher birth weight and length (-0.40 vs. -0.67 SDS, and -0.19 vs. -0.51 SDS, respectively). It is well known that, despite GH being the key hormone for maintaining postnatal growth, it plays a negligible role in prenatal growth (45, 46); even in cases of congenital GH deficiency — excluded from this study due to their specific characteristics — birth length is not affected (47).

Compared to the dGHD group, children with SUS had a lower MPH (-1.20 vs. -0.65 SDS) and a lower difference between final height and MPH (-0.05 vs. -0.34 SDS). Children with ISS had an even lower MPH (-1.33 SDS) and a lower difference between final height and MPH (-0.13 SDS). This suggests that at least some SUS and ISS patients may fall under the category of ‘familial short stature’ (FSS), suggesting that an autosomal dominant form of short stature might coexist in this subgroup of patients (48). It is often observed that children with short stature, initially diagnosed with GHD, may actually have genetic variants associated with growth, but rarely involving GH secretion or function (49, 50). In fact, the GH-IGF-1 axis has recently been found not to be the sole determinant of chondrogenesis and growth, which are also greatly influenced by a complex interplay of hormonal, paracrine, extracellular, and intracellular factors (51). The low GH peak observed during stimulation tests in SUS could thus be viewed as an epiphenomenon rather than the underlying cause. Indeed, the SUS category may include a variety of patients whose short stature, rather than being caused by definite GH deficiency, may be due, for example, to growth plate disorders. The latter are frequent amongst children with a “GHD” diagnosis, and have an excellent response to rhGH therapy (50). It is, in fact, also known that response to rhGH treatment is not specific for GHD, as children with monogenic causes of short stature different from GHD and even children with ISS experience height gain with replacement therapy (50, 52). The role of genetic testing in shaping the modern diagnostic approach to pediatric short stature is undeniable (53–55). Unfortunately, the period during which the KIGS registry was utilized does not reflect the current era, with its availability of advanced panels and clinical tests.

Several limitations of this study should be noted.

First, as an observational study, this study has several potential limitations. Since this is a retrospective cohort study, without randomization, confounding and biases cannot be entirely eliminated, and the determination of causation is limited. Enrollment bias is possible, though sites were expected to enroll all eligible patients treated with rhGH. Follow-up bias over the duration of KIGS is a potential limitation as well; estimates presented in the manuscript are based on patients with non-missing data. Furthermore, only patients who remained in the registry until reaching NAH were included, whereas dropouts—who may have had less favorable outcomes—were not captured. Therefore, our results should be interpreted as conditional on persistence in follow-up until NAH.

Additionally, although the dataset includes a large number of patients, data on certain variables (such as IGF-1 levels, e.g.) were incomplete for some individuals, which may affect the accuracy of our results and, to some extent, cause some overlap between the three groups. Furthermore, we did not have information on the use of sex steroid priming before GH stimulation tests, nor on the presence of ADHD or other neurodevelopmental disorders, or the use of psychoactive medications. These unmeasured factors may have influenced GH test responses in some children. In addition, our analyses applied Italian diagnostic criteria and cut-offs to all patients in the KIGS database. While this approach ensured consistency across the cohort, it may not fully reflect the heterogeneity arising from country-specific diagnostic and treatment practices. Lastly, it must be acknowledged that the lack of genetic analyses does not allow us to establish a definitive pathogenetic mechanism for short stature in all categories of patients. All these limitations should be considered when interpreting our findings.

However, this is the first large (almost 4,000 children who started rhGH treatment in pre-puberty) multi-centric study comparing dGHD, SUS, and ISS children who underwent treatment with rhGH, and, even without genetic confirmation, it shows that the three groups have distinct clinical, auxological, and laboratory characteristics. Furthermore, the extended timeframe of the study has allowed long follow-up times and thus identification of predictive factors for height SDS at the end of rhGH treatment across the three categories of patients.

These results underscore the need to reconsider the use of the GHD label for children without clear-cut evidence of deficiency. It is not merely a semantic issue to use the definition of SUS instead of “idiopathic GHD”. Firstly, categorizing a case as idiopathic GHD still results in a diagnosis of GHD, even without identifying a specific cause. This diagnosis would be based on stimulation tests, which are not highly reliable, and on characteristics inconsistent with true GHD (e.g., normal IGF-1 levels, normal BMI, and a lower target height). Moreover, the follow-up approach differs significantly, as in true GHD, the risk of persistent deficiency and the development of other pituitary deficits is much higher (56). In our opinion, this study further emphasizes the need for a correct terminology when performing the differential diagnosis of short stature, which must be supported by clinical, auxological, and laboratory findings but should ultimately be confirmed through genetic testing. Indeed, even though short stature is unlikely to have a monogenic origin, knowledge on its genetic causes is continuously improving, with rates of genetic diagnosis in “idiopathic” short stature as high as 46% with whole exome sequencing (WES) analysis (57). While it may be argued that such genetic testing may be an economic burden, so are stimulation tests and rhGH therapy, which should thus be offered to children who can most benefit from them (7). Furthermore, retaining these children under the GHD umbrella risks inappropriate expectations, unnecessary follow-up for pituitary dysfunction, and a misleading clinical narrative for families. Our findings strengthen the case for adopting more accurate terminology and classification strategies—grounded in biology, not just GH peak thresholds—in line with modern principles of precision medicine. We hope that in the future this may aid in offering both clinicians and patients’ families a more accurate prediction of current and future height with and without hormone supplementation.

## Conclusion

In conclusion, this study highlights the importance of classifying children with growth disorders into distinct diagnostic categories (dGHD, SUS, and ISS) to better understand their response to rhGH therapy and predict their growth outcomes. Our findings support the clinical value of rhGH treatment across different diagnostic groups, including patients with GHD of unknown etiology. All three groups—SUS, dGHD, and ISS—showed a positive response to therapy, with a general reduction in the proportion of individuals presenting with short stature at NAH. Although children with dGHD and SUS had almost the same response to rhGH in terms of NAH, they differ in baseline characteristics and may have different follow-up. When an identifiable anatomical or functional cause for GHD is not found, we believe that genetic testing should be considered in all children with SUS.

## Data Availability

The data analyzed in this study is subject to the following licenses/restrictions: The KIGS registry (Pfizer International Growth Database) is a property of Pfizer. Requests to access these datasets should be directed to Martin.Carlsson@pfizer.com.
